# Breaking down the evidence for bevacizumab in advanced cervical cancer: past, present and future

**DOI:** 10.1186/s40661-015-0015-0

**Published:** 2015-09-21

**Authors:** Victor Rodriguez-Freixinos, Helen J. Mackay

**Affiliations:** Division of Medical Oncology and Hematology, Princess Margaret Hospital, University of Toronto, 610 University Avenue, Toronto, Ontario M5G 2 M9 Canada

**Keywords:** Angiogenesis, Bevacizumab, Recurrent and metastatic cervical cancer, Target therapy, Human papilloma virus

## Abstract

Despite the introduction of screening and, latterly, vaccination programs in the developed world, globally cervical cancer remains a significant health problem. For those diagnosed with advanced or recurrent disease even within resource rich communities, prognosis remains poor with an overall survival (OS) of just over 12 months. New therapeutic interventions are urgently required. Advances in our understanding of the mechanisms underlying tumor growth and the downstream effects of human papilloma virus (HPV) infection identified angiogenesis as a rational target for therapeutic intervention in cervical cancer. Anti-angiogenic agents showed promising activity in early phase clinical trials culminating in a randomized phase III study of the humanized monoclonal antibody to vascular endothelial growth factor (VEGF), bevacizumab, in combination with chemotherapy. This pivotal study, the Gynecologic Oncology Group protocol 240, met its primary endpoint demonstrating a significant improvement in OS. Bevacizumab became the first targeted agent to be granted regulatory approval by the United States Food and Drug Administration for use alongside chemotherapy in adults with persistent, recurrent or metastatic carcinoma of the cervix. This review outlines the rationale for targeting angiogenesis in cervical cancer focusing on the current indications for the use of bevacizumab in this disease and future directions.

## Introduction

Following the introduction of population based screening, the incidence of cervical cancer has been declining in the developed world a trend that is expected to continue with the increased availability and implementation of HPV vaccination programs. Globally, however, cervical cancer remains a major health issue and is the third most common cancer affecting women with 85 % of the diagnoses and 88 % of deaths due to this disease occurring in resource poor regions of the world [1]. Even within the United States (US), despite the availability of screening programs, in 2015 over 12,000 women will be diagnosed with cervical cancer with approximately 4000 women expected to die from their disease. Furthermore, between 2000 and 2012 the proportion of women diagnosed with stage IV cervical cancer in the US rose [2]. Advanced cervical cancer disproportionately affects women from lower socio-economic groups, those who are under or uninsured, women of African American or Hispanic ethnicity and those from medically underserved communities [3].

Early-stage cervical cancer is a potentially curable disease either by surgery (for those diagnosed with International Federation of Gynecology and Obstetrics (FIGO) stage IA/B1 disease) or by a combination of low dose chemotherapy administered concurrently with radiotherapy followed by intracavitary brachytherapy. For those not suitable for local control, who recur or who are diagnosed with metastatic disease outcomes are poor with 5-year survival rates between 5 and 15 % [4]. In this setting any treatment is palliative and the goals of care are to prolong survival but also, and perhaps more importantly, to maintain and/or improve quality of life (QoL). A number of first line, cisplatin based, doublet, combination chemotherapy regimens have been investigated in prospective randomized clinical trials conducted by the Gynecologic Oncology Group (GOG). These culminated in GOG 204, a four-arm study that compared cisplatin in combination with paclitaxel, vinorelbine, gemcitabine or topotecan [5]. Outcomes were similar in all arms with a non-significant trend in favor of cisplatin/paclitaxel (overall survival (OS) 12.9 months) compared to the other three arms (OS 10–10.3 months) and similar overall response rates (ORR). In a further randomized phase III clinical trial conducted by the Japanese GOG (JGOG) carboplatin in combination with paclitaxel was found to be non-inferior to cisplatin/paclitaxel [6]. For women with poor prognostic features including poor performance status, prior treatment with chemoradiation or recurrence within 1 year, response duration can be short at less than 6 months in some cases. Response to treatment is also influenced by the site of recurrence with disease control in previously irradiated areas proving particularly challenging [7]. There are no standard of care second line options for these women when their cancer progresses. New therapeutic approaches are, therefore, urgently required. However, the cervical cancer patient demographic also poses unique challenges in terms of sustainable drug development where cost effectiveness and access to new treatments for those in need are key issues. Furthermore, conducting clinical trials in this patient group can also pose difficulties as women diagnosed with advanced cervical cancer frequently come from sections of society where, historically, engagement in clinical research has been low.

Targeting angiogenesis is one of the most promising therapeutic strategies to emerge in recent years in the treatment of cervical cancer. Angiogenesis is a critical process in cervical carcinogenesis and tumor progression. Following the publication in 2014 of the randomized phase III study GOG 240, the US Food and Drug Administration (FDA) approved the first anti-angiogenic agent, bevacizumab (Avastin, Genentech/Roche), in combination with chemotherapy for use in women with advanced cervical cancer [8]. This article will review the rationale for studying anti-angiogenic therapy in cervical cancer, focus on the clinical use of bevacizumab and finally highlight potential future directions.

## Review

### Angiogenesis in cervical cancer and rationale for targeting

#### Angiogenesis

Angiogenesis is a physiologic and highly ordered process that involves the regulation of multiple signaling pathways and requiring interaction between different cell types, including endothelial cells, stromal cells (fibroblasts), and their interaction with the extracellular matrix, cytokines and growth factors, which leads to the effective formation of new blood vessels. Hypoxia and the mechanisms that mediate hypoxic response are key drivers of physiologic angiogenesis. Under hypoxic conditions expression of hypoxia inducible factor, (HIF- 1α) is induced in endothelial cells, resulting in VEGF-A, and vascular endothelial growth factor receptor 2 (VEGFR-2) expression [9]. Although numerous proangiogenic factors have been described there is universal agreement that the VEGF family of ligands, (VEGF-A, to -D and placental growth factor [PLGF]) and their associated receptor tyrosine kinases (VEGFR)-1, 2 and 3 are the most important regulators of angiogenesis. VEGF-A, usually referred to as VEGF, binds to VEGFR-1 and VEGFR-2; the stimulation of endothelial cell mitogenesis and vascular permeability is mediated by its interaction with VEGFR-2 [10]. PLGF and VEGF-B selectively bind to VEGFR-1 and stimulate vessel growth and maturation and recruit proangiogenic bone marrow-derived progenitors [11, 12]. VEGF-C and VEGF-D primarily interact with VEGFR-3 stimulating lymphangiogenesis [13]. Other crucial steps in physiologic angiogenesis involve the recruitment of pericytes. Pericytes, recruited primarily by platelet-derived growth factor (PDGF), secreted by endothelial cells, are essential for the stabilization, maturation and support of new vessels [14]. Angiopoietins (Angs) 1 and 2 are expressed on the surface of pericytes and are ligands of the endothelial cell receptor Tie-2. Thus, the angiopoietin/Tie pathway is involved in the stability of mature vessels and proliferation of endothelial cells. However, the contribution of Ang-1 and Ang-2 to the angiogenesis process is distinct. Ang-1 functions as a Tie2 receptor agonist when it binds to TIE-2 receptors expressed on the surface of endothelial cells, maintaining the integrity of existing vessels. In contrast, Ang-2 is mainly secreted by endothelial cells at sites of active vascular remodeling. Ang-2 acts antagonistically to Ang1, promoting sprouting angiogenesis facilitating the effects of VEGF [15, 16], whilst VEGF also upregulates Ang-2 in endothelial cells [17].

Many cancers exploit aberrant angiogenic mechanisms to stimulate tumor growth and metastasis. Tumor angiogenesis was established as a potentially attractive therapeutic target for the treatment of cancer with the publication of Folkman’s hypothesis in 1971 [18]. Angiogenesis is required for tumor growth beyond 1-2 mm^3^, when the tumor demand for oxygen and nutrients surpasses the local supply and the hypoxic microenvironment, through the expression of HIFs leads to the activation of angiogenesis. Tumor related angiogenesis, in contrast to physiologic angiogenesis, leads to a more disorganized vasculature, which is also more permeable, limiting the delivery of drugs to tumor cells. Anti-angiogenic agents have been shown to transiently ‘normalize’ the tumor vasculature, resulting in an increased delivery of oxygen and drugs into the tumor microenvironment [19]. Many cancers induce VEGF-A expression promoting the formation of new tumor blood vessels, rapid tumor growth, and facilitation of metastatic potential [20]. Other mechanisms also contribute to tumor related angiogenesis, such as overexpression of VEGF receptors, especially VEGFR-1. Several multi-target tyrosine-kinase inhibitors (TKIs) of VEGFR have recently been evaluated showing encouraging results [21]. In addition, constitutive activation in a number of oncogenes such as *ras*, *PI3k* and *src*, or the loss of tumor suppressor function, for example through mutations in the tumor suppressor gene von Hippel Lindau which enhances the activity of HIF1α, have the capacity to induce proangiogenic factors and growth factors, promoting tumor angiogenesis [22–24].

VEGF-A potentiates proliferation of endothelial cells by activating the C-Raf-MAPK/ERK kinase signaling pathway [25]. Furthermore, there is interplay between other proangiogenic pathways, which are upregulated in tumors. These include Angs, fibroblast growth factor (FGF)/fibroblast growth factor receptor (FGFR), PDGF/platelet-derived growth factor receptor (PDGFR), hepatocyte growth factor (HGF)/MET and the PI3K/Akt/mTOR signaling pathways. The Ang–TIE2 pathway is of particular interest, as Ang-1 and 2 are upregulated in many cancer subtypes. Research on this signaling system has also provided evidence on the role of pericyte cells, which secrete ang-1 and express PDGF receptors, and explains the anti angiogenic action of some of the multitargeted TKI inhibitors [26, 27]. The PDGF family consists of PDGF-A to -D polypeptide homodimers and the PDGF-AB heterodimer ligands and their binding tyrosine kinase receptors, PDGFR-α and –β. Aberrant activation of this pathway is implicated in pericyte recruitment to vessels; secretion of proangiogenic factors; stimulation of endothelial cell proliferation, and promotion of lymphangiogenesis among others [28]. The FGF/FGFR family compromises a total of 23 members, 18 of which function as ligands for four receptor tyrosine kinases (FGFR-1 to −4), regulating normal cell growth and differentiation and angiogenesis [29]. Overexpression of FGF, mainly FGF1 and FGF2, and FGFR contribute to different mechanisms, such as activating mutations, gene amplification and translocations, among others, leading to enhanced angiogenesis through the stimulation and release of other proangiogenic factors [30]. In addition, a collaborative interplay between FGF and VEGF signaling has also been demonstrated to be important for angiogenic and metastatic processes [31]. The inhibition of these alternate pathways (PDGF, FGF) may mediate resistance and potentiate VEGF inhibition, supporting a multitargeted approach inhibiting both VEGFR and PDGFR [32]. The HGF/MET binding also mediates tumor angiogenesis and growth in a variety of epithelial malignancies. The HGF/MET axis is responsible for the cell-scattering phenotype and increases angiogenesis by direct activation of endothelial cells or via downstream stimulation of pro-angiogenic pathways, including PI3K/Akt and Src and production of proangiogenic factors, such as VEGF [33–35]. The PI3K/Akt/mTOR cascade is also involved in angiogenesis through the interaction of the mTOR complex 1 and 2 with the VEGF pathway, and moreover, Akt has shown importance for endothelial cell survival [36, 37].

Greater understanding of these pathways continues to provide valuable insights into the molecular mechanisms that underlie tumor angiogenesis and provide a foundation for the development of novel anti-angiogenic therapeutic strategies.

#### Angiogenesis in cervical cancer

**Fig. 1:**
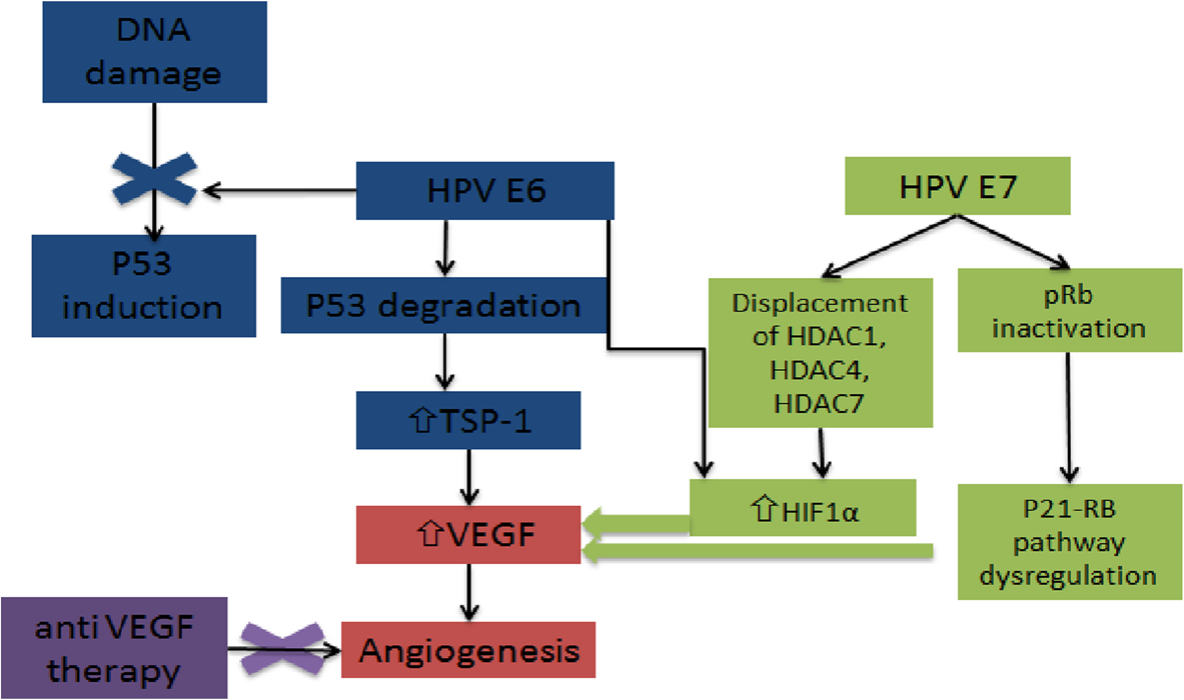
Tumor Hypoxia and Viral Oncogenes Drive Angiogenesis. Abbreviations: HPV: Human papillomavirus; pRb: retinoblastoma gene product; HDAC1, 4, 7: Histone deacetylases 1, 4, 7; TSP-1: Thrombospondin-1; HIF-1α: hypoxia-inducible factor 1 alpha; VEGF: Vascular endothelial growth factor

High-risk HPV subtypes 16 and 18 (although other subtypes have also been implicated) are responsible for approximately 70 % of invasive cervical cancers [38]. Emerging data suggest that viral integration into the host cell genome results in overexpression of a number of host genes, which are potential drivers of carcinogenesis [39]. However, the HPV oncoproteins E5, E6, and E7 are the primary viral factors responsible for initiation and progression of cervical cancer. E6, E7 and to a lesser extent E5 play key roles in upregulating angiogenesis through the VEGF pathway through their effects on p53 degradation, HIF-1α and inactivation of retinoblastoma protein (pRb). HPV E6 promotes p53 ubiquitination and degradation after E6-p53 binding. Degradation of p53 promotes angiogenesis by down regulating thrombospondin-1 and by increased production of VEGF. HPV E7 results in abrogation of pRb function resulting in p21-RB pathway dysregulation thereby increasing VEGF. In addition, HPV E6 (in a p53 independent manner) and E7 also enhance the induction of HIF-1α, thus increasing VEGF through a second mechanism [40–43] (Fig. 1).

Over the past decade, the relationship between HPV-16 and 18 associated cervical tumors, hypoxia, markers of tumor angiogenesis and prognosis has emerged. Initial descriptions of high risk HPV related premalignant cervical lesions seen on colposcopy included atypical angiogenic proliferation along the basement membrane and suggested a role for angiogenesis in the transition from premalignant lesions to invasive cervical carcinoma. Microvessel density (MVD) has been reported to increase with the grade of pre-malignant lesions [44]. High intratumoral microvessel density (MVD) in cervical cancer has been associated with poorer prognosis, advanced stage at presentation, and greater risk of nodal involvement [45]. However, this data is controversial with some studies showing a poor prognosis, others a better prognosis and some no effect on outcome. In an analysis performed on tumor specimens from the phase III study GOG 109 [45], which investigated the addition of cisplatin chemotherapy to adjuvant radiation following radical hysterectomy, MVD was an independent prognostic marker for improved progression-free survival (PFS) and OS [hazard ratio (HR) = 0.36, 95 % CI: 0.17–0.79, *p* = 0.010]. In an ad hoc analysis of GOG 109 [46], specimens were assessed for expression of markers of tumor angiogenesis including VEGF, TSP-1 (anti-angiogenesis factor), cluster of differentiation 31 (CD31) and CD105 (tumor-specific endothelial marker). CD31 was used in the GOG 109 analysis to measure MVD and predicted for a good outcome. In contrast, the presence of CD105-positive vessels in cervical cancer samples has shown an association with risk of lymph node metastasis, and worse PFS and OS [47]. The differences in outcome observed in these studies may relate to the method used to study MVD. Some markers such as CD31, used in GOG109, may reflect “good angiogenesis”, with CD31 positive endothelial cells exhibiting organized vasculature, potentially leading to well vascularized and oxygenated tumors, leading to better outcomes, whilst other markers such as CD105 may indicate a more disordered endothelial structure resulting in poorer outcomes. In addition, analysis of VEGF has shown increased VEGF expression in cervical intraepithelial neoplasia grade III and squamous cell carcinoma when compared with control cervical tissue. In the cervical cancer samples higher VEGF levels were associated with advanced stage disease, increase risk of nodal metastasis, and worse PFS and OS [48]. In cervical carcinomas, elevated serum VEGF has been identified as a poor prognostic factor [49, 50].

Angiogenesis plays a pivotal role, not only in initiation of cervical cancer, but also in proliferation and progression of the disease, hence targeting angiogenesis has emerged as a rational therapeutic approach.

### Bevacizumab in advanced and recurrent cervical cancer

Improving the limited success achieved with traditional cytotoxic chemotherapy in patients with recurrent and metastatic cervical cancer represents a critical unmet medical need. Metastatic cervical cancer patients present a number of challenges including: disease related complications (obstructive uropathy, bleeding); impact of prior therapies (particularly when recurrence occurs in a previously irradiated field), poor performance status and frequent psychosocial issues.

**Fig. 2:**
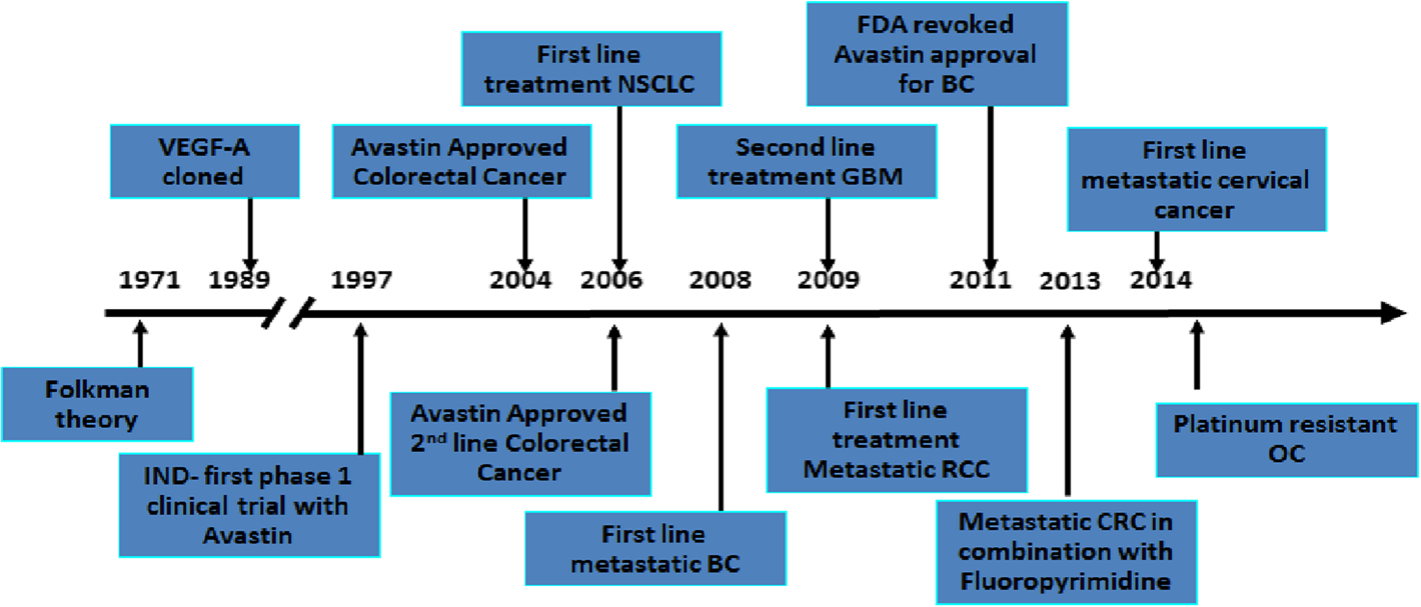
Indications granted FDA regulatory approval for Bevacizumab for solid tumors treatment. Abbreviations: VEGF-A: vascular endothelial growth factor; IND: Investigational New Drug Application; NSCLC: non-small cell lung cancer; BC: breast cancer; RCC: renal carcinoma; CRC: colorectal cancer; OC: ovarian cancer

Bevacizumab is a recombinant humanized monoclonal IgG1 antibody directed against VEGF-A which blocks signal transduction through VEGFR-1 and 2 associated pathways. In preclinical models bevacizumab suppressed VEGF-induced tumor growth and reduced tumor MVD. Bevacizumab appeared to normalize primitive tumor vasculature, leading to an increase in tumor oxygenation and potentially enhancing delivery of cytotoxic agents thereby potentiating their efficacy [51]. Bevacizumab has shown clinical activity in different solid tumor types resulting in approval by the FDA for treatment of metastatic colorectal cancer, non-small cell lung cancer, renal cell carcinoma, glioblastoma multiforme and ovarian cancer (Fig. 2).

Wright and colleagues initially reported the clinical utility of bevacizumab in the treatment of persistent or recurrent cervical cancer patients. This small retrospective analysis showed a meaningful clinical benefit rate of 67 % in a heavily pretreated patient population (median of 3 prior regimens), when bevacizumab was combined with chemotherapy [52]. These results catalyzed a phase II trial conducted by the GOG (GOG 227C), which aimed to determine the efficacy and toxicity profile of single agent bevacizumab in advanced cervical cancer patients. This study demonstrated encouraging clinical activity which compared favorably with historical single agent cytotoxic phase II studies in similar previously treated patient populations [53]. Among 46 evaluable patients, the ORR was 11 %, with median response duration of 6.21 months (range, 2.83 to 8.28 months), and a median PFS and OS of 3.4 months (95 % CI: 2.53–4.53 months) and 7.3 months (95 % CI, 6.11–10.41 months), respectively. The 6-month PFS rate was 24 %. In this study, almost 83 % of patients had received prior pelvic radiation and 74 % had received at least one prior cytotoxic regimen for recurrent disease (74 %). Bevacizumab was generally well tolerated, fistula occurring in only 2.17 % of patients. Following on from GOG 227C, the combination of bevacizumab with platinum-based chemotherapy was investigated in a further phase II clinical trial. Twenty seven women undergoing first line treatment for locally advanced or recurrent disease received bevacizumab 15 mg/kg combined with cisplatin and topotecan administered on a 21-day cycle. Although the results in median PFS and OS were encouraging (7.1 months and 13.2 months respectively), the toxicity reported from the combination was significant with grade 3–4 hematologic toxicity being common (thrombocytopenia 82 %, anemia 63 %, and neutropenia 56 %) and a significant fistula rate of 26 % [54].

**Fig. 3:**
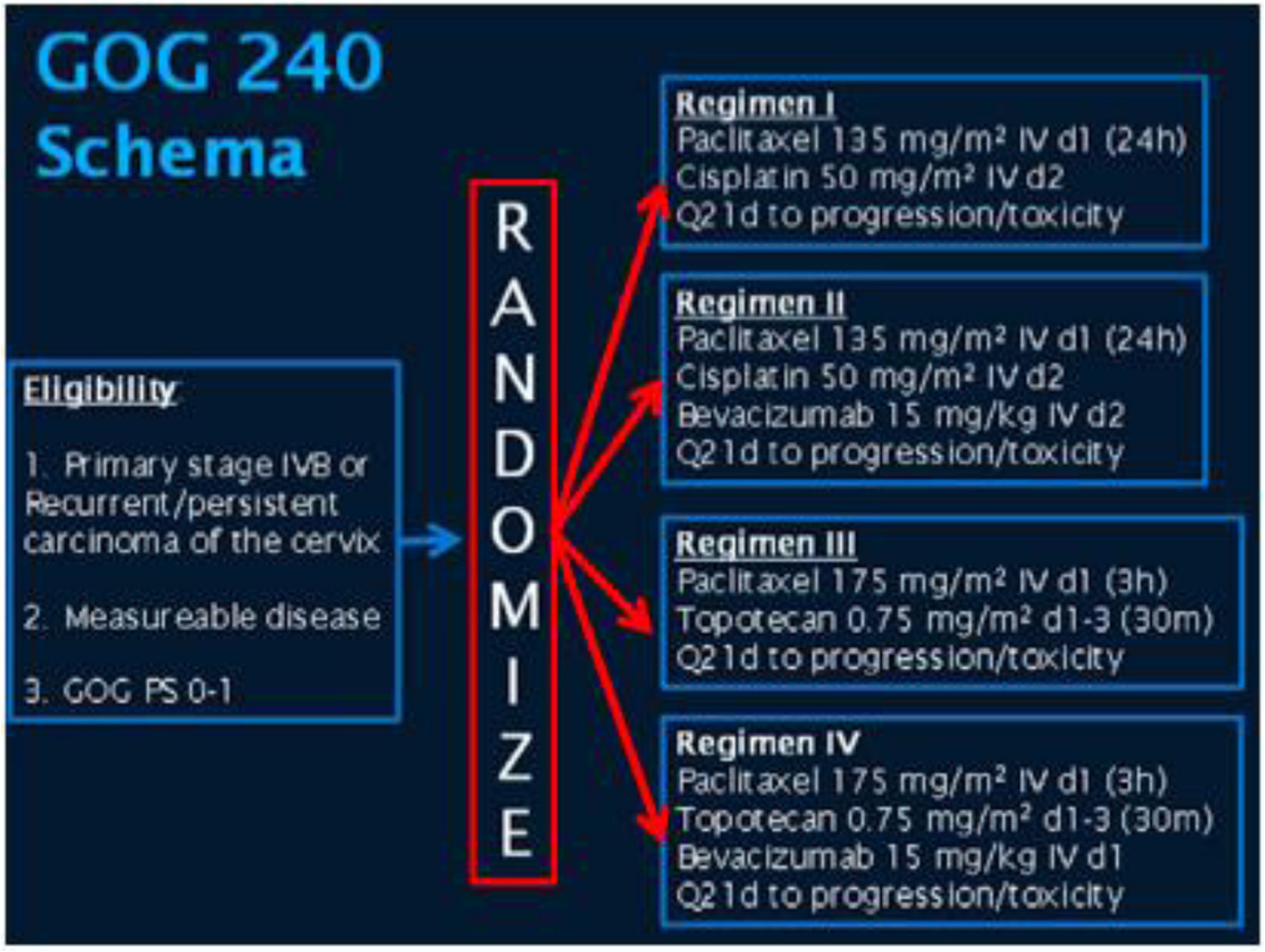
GOG 240 study design. Abbreviations: GOG, Gynecologic Oncology Group; PS, performance status; m^2^: square meters, mg: milligram, IV: intravenous, Kg: kilogram. Figure 3 is from *I. Diaz*-*Padilla et al. Critical Reviews in Oncology*/*Hematology 85* (*2013*) *303*–*314* [77] *and is used with permission*

Following on from the promising activity observed in early phase clinical trials, a four-arm prospective, randomized clinical trial, GOG 240, was conducted. The aim of GOG 240 was to demonstrate whether the addition of bevacizumab to chemotherapy lead to an improvement in OS. In addition ORR, PFS, toxicity and health related Quality of Life (HR QoL) end points were also explored. GOG 240 had a 2 × 2 factorial study design that involved randomization to both the standard cisplatin and paclitaxel arm and to a non-platinum containing regimen, paclitaxel and topotecan, with or without Bevacizumab 15 mg/kg intravenously every 21 days (Fig. 3). In the modern era, exploration of a non-platinum based combination was of interest as many patients receive cisplatin in combination with radiotherapy for their definitive frontline treatment; hence cisplatin may be less effective than previously reported following the introduction of chemotheradiotherapy as a standard of care. Stratification factors included stage IVB vs. recurrent/persistent disease, PS 0–1 and prior concomitant Cisplatin and radiation. Treatment was continued until disease progression (PD), unacceptable toxicity or complete response (CR). In addition, archival diagnostic tissue was collected for correlative studies.

GOG 240 was activated on April 9, 2009 reaching target accrual on January 2, 2012, for a total of 452 patients. Sample size calculation was based on increasing the median OS from 12 to 16 months, detecting with 90 % power, a reduction in the risk of death of at least 30 %, with the one-sided type I error rate limited to 2.5 % for each regimen. Over 220 patients were treated with each of the chemotherapy backbones (225 chemotherapy alone, 227 chemotherapy plus bevacizumab). Clinical characteristics were well distributed between groups receiving the 2 backbones: median age of enrolled patients was 49 years; the majority of patients had squamous cell cancer (70 %) with 20 % having adenocarcinomas. The majority of patients had recurrent disease (73 % chemotherapy arm and 70 % chemotherapy plus bevacizumab arm). The rate of persistent disease was 11 % in both arms and 16 % of patients in each arm presented with advanced disease at diagnosis. The proportion of prior platinum chemotherapy in combination with radiotherapy was also well-balanced between each arm (74 % and 75 % in the chemotherapy and the investigational arm respectively, *p* = 0.666). 55 % of patients had locally recurrent pelvic disease after chemoradiotherapy. Notably, the majority of patients in each chemotherapy group had a PS of 0 (PS 0–1 required for enrollment).

A pre-planned interim analysis after 174 deaths to determine futility/superiority was conducted on February 6, 2012 and presented at the Society of Gynecologic Oncology (SGO) meeting in 2013 [55]. This demonstrated that the topotecan-paclitaxel arm was not superior or inferior to the cisplatin-paclitaxel arm (median OS 15 *vs*. 12.5 months respectively, HR 1.20; 95 % CI: 0.82–1.76). Following a second analysis, with a median follow-up of 20.8 months the National Cancer Institute’s (NCI) Data Safety Monitoring Board (DSMB) recommended ending the trial and also, due to the data’s potential to alter the standard of care, that the results were released into the public domain [56]. The study demonstrated a significant improvement in OS for the addition of bevacizumab to chemotherapy compared to chemotherapy alone (17 months *versus* 13.3 months respectively; HR = 0.71; 95 % CI: 0.54–0.95; *p* = 0.0035). In addition, the benefit of bevacizumab was reported for both chemotherapy regimens—cisplatin-paclitaxel ± bevacizumab median OS 14.3 *vs*. 17.5 months (*p* = 0.03) and topotecan-paclitaxel ± bevacizumab-median OS 12.7 *vs*. 16.2 months (*p* = 0.08). The median PFS in the bevacizumab group was 8.2 months compared with 5.9 months in the chemotherapy alone group (HR 0.67; 95 % CI, 0.54–0.82; *p* = 0.0002). Response rate also was higher in the bevacizumab group 48 *vs*. 36 % (*p* = 0.008). The exploratory subgroup analysis suggested that the effect of bevacizumab was consistent across multiple prognostic subgroups, and that prior platinum exposure or recurrent disease in the pelvis after prior radiation did not preclude benefit from bevacizumab. These data, published in the New England Journal of Medicine [57], represented the first time a targeted agent showed improvement in OS in patients with cervical cancer. Recent planned subgroup analyses presented in abstract form only, suggested that the addition of bevacizumab was associated with a greater likelihood of CR within the irradiated pelvis (61 %, *N* = 11) compared to chemo alone (39 %, *N* = 7), and that achieving CR (44/452 patients (9.7 %) is associated with prolonged OS (OS 39.3 months while median OS for patients with CR on the cisplatin–paclitaxel–bevacizumab arm has not been reached) [58]. Previously described poor prognostic factors including African American ethnicity, PS, measureable disease within the pelvis, prior cisplatin, and short progression-free interval were also prognostic in GOG 240. However, the investigators questioned their utility at guiding whether to add bevacizumab as high-risk patients did appear to benefit [59].

The addition of bevacizumab to chemotherapy did, however, result in increased toxicity, notably; increased risk of fıstula formation and perforation of the gastrointestinal and genitourinary tracts (10.9 vs. 1 %, *p* = 0.002), grade 2 hypertension (25 vs. 2 %, p < 0.001), grade 4 neutropenia (35 vs. 26 %, *p* = 0.04), and thromboembolism (8 vs. 1 %, *p* = 0.001). Gastrointestinal and genitourinary bleeding grade 3–4 was uncommon (2 % vs <1 %, *p* = 0.37 and 3 % vs <1 %, *p* = 0.12, respectively), and clinically relevant central nervous system bleeding did not occur. Fistulae and perforations appeared to occur exclusively in patients who had undergone prior pelvic radiotherapy (reported in abstract form only) [60]. A better understanding of patients at risk is required if we are to minimize fistula/perforation rates in the clinic and adequately advise patients regarding the level of risk. In addition, although differences in HRQoL, assessed using the Functional Assessment of Cancer Therapy—Cervix Trial Outcome Index scale (FACT-Cx TOI scale), did not reach statistical significance on average HRQoL was 1.2 points lower in the bevacizumab containing treatment arm (99 % CI, −4.1 to 1.7; *p* = 0.30) [61].

On August 14, 2014, under the FDA Priority Review program [62] bevacizumab in combination with chemotherapy (both study arms) was granted regulatory approval in the US for treatment of cervical cancer. Following the FDA approval, the National Comprehensive Cancer Network (NCCN) upgraded cisplatin-paclitaxel-bevacizumab to category 1 in August 2014 and listed topotecan-paclitaxel-bevacizumab as category 1 in September 2014 [63]. The final analysis from GOG 240 has confirmed that benefits obtained from the addition of bevacizumab are sustained after 348 events and with a median follow-up of 50 months; bevacizumab-containing regimens continue to demonstrate a significant improvement in OS over chemotherapy alone: 16.8 vs 13.3 months (HR 0.765, 95 % CI: 0.62, 0.95;*p* = 0.0068) [64]. However, survival in the control arms of GOG240 was greater than in previous studies and potentially reflects the higher PS of the clinical trial patient population. How outcome and toxicity translate in the broader non trial patient population is awaited a further area where “real world data” will better inform future clinical practice.

Whilst the data from GOG 240 resulted in a change to the standard of care not all women benefited and that benefit was relatively short lived. Identification of predictive biomarkers both for response and for toxicity is desirable if we are to optimize the use of this drug. Initial reports from correlative studies evaluated the impact of pretreatment circulating tumor cells (CTCs) on OS showing a correlation between high pretreatment CTC counts, and greater declines of CTC during treatment, with lower risk of death (HR 0.87; 95 % CI 0.79, 0.95) upon addition of bevacizumab [65]. Data from this and from other correlative studies are required and validation is essential if predictive biomarkers are to become clinically useful. There are potentially opportunities to explore predictive biomarkers across tumor types and data sets which may benefit a larger number of patients, particularly in relation to prediction of toxicity.

Whilst the addition of bevacizumab to chemotherapy has become a new standard of care for women in resource rich communities it remains inaccessible to those at greatest need. The cost implications and generalizability of incorporating bevacizumab in poorly resourced countries and communities is a significant issue, however questions around cost-effectiveness have also risen even in resource rich regions. An initial cost-effectiveness analysis reported by Phippen et al. showed an incremental cost-effectiveness ratio (ICER) of $155 K/quality adjusted life year (QALY) [66]. However, an updated analysis using a Markov decision tree model that incorporated the final OS and toxicity data (improvement of 3.9 months and fistula rate of 8.6 %) showed the cost of the addition of bevacizumab was $53,784 compared to $5688 for the chemotherapy alone arm. Thus, the addition of bevacizumab represents an increase of 13.2 times the cost for chemotherapy alone, adding $73,791 per 3.5 months of life gained and an incremental ICER of $21,083 per month of added life, mostly due to the cost of bevacizumab rather than related with the management of related toxicity [67]. Further exploratory analysis also suggested that adding Bevacizumab would become cost-effective, with a significant decline in the ICER, by either reducing the dose of bevacizumab from 15 to 7.5 mg/kg, or diminishing the costs of bevacizumab. Whether the benefit conferred by bevacizumab is worthwhile or not from a cost-effectiveness perspective, remains a societal and clinical dilemma. Further cost-effective analyses based on real world experience, are warranted. In addition the introduction of generic drugs into the market may in time reduce the cost of targeting angiogenesis using this approach. However, this will not remove the need to advocate on a global level for accessible health care for our most economically vulnerable patient populations.

### Summary and future directions

**Fig. 4:**
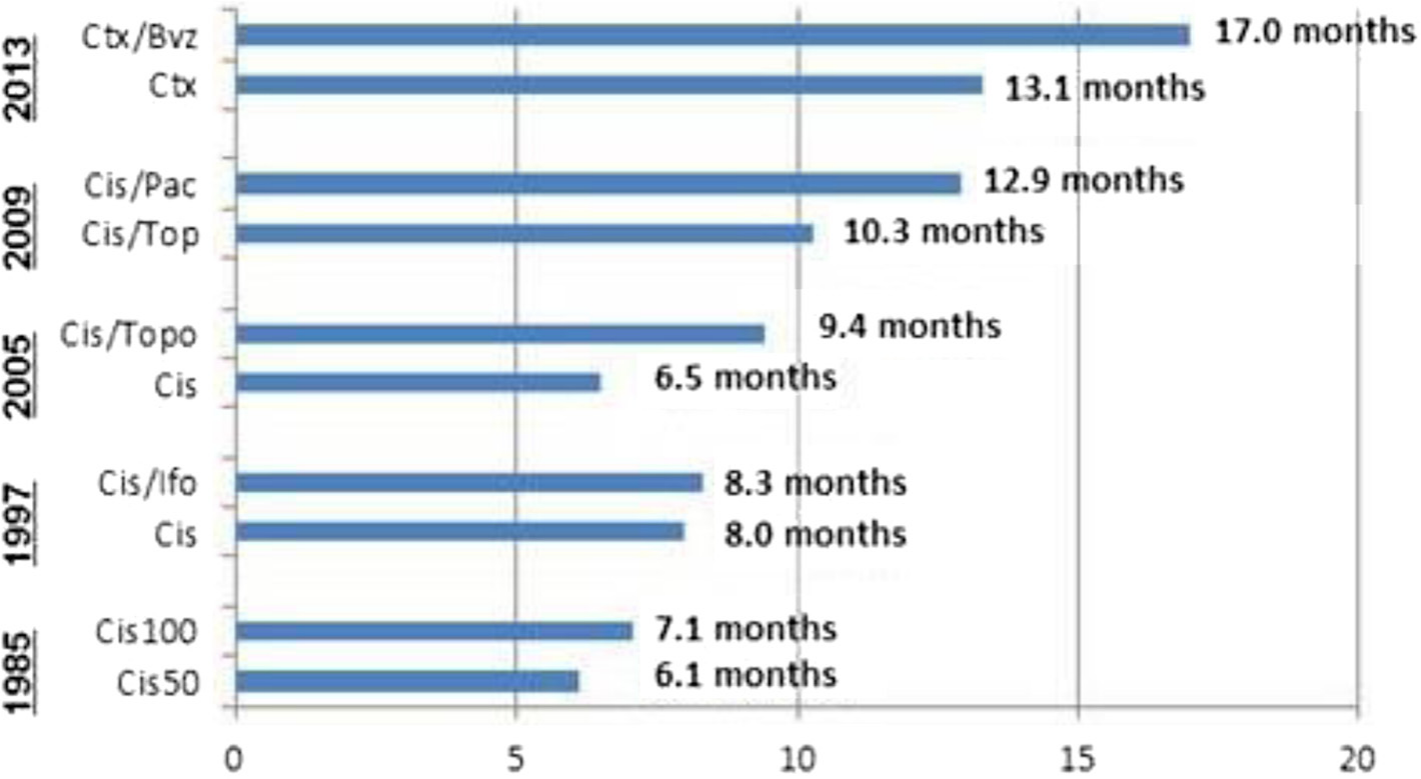
Improvement in overall survival in advanced cervical cancer. GOG phase 3 trial experiences. Abbreviations: Bev: bevacizumab; Cis: cisplatin; Ctx: chemotherapy; Ifo: ifosfomide; OS: overall survival; Pac, paclitaxel; Topo: topotecan

Angiogenesis is central to cervical cancer development and progression. Publication of GOG 240 showing a significant improvement in OS, PFS and ORR, without a concomitant deterioration of HRQoL, demonstrated proof of concept concerning integration of anti-angiogenesis therapy for advanced cervical cancer patients, and represents a practice-changing clinical trial. The significance of 3.7 months improvement in OS is most clear when placed in context with prior clinical trials in this setting (Fig. 4) [57, 68–71], (Table 1) [5, 6, 57, 70, 71]. Targeting angiogenesis is therefore a successful strategy that should be further investigated in the next generation of clinical trials.

**Table 1:** Comparison of GOG phases 3 randomized clinical trials for women with recurrent or advanced cervical cancer. GOG Protocols 169, 179, 204, 240 and JGOG 0505

	GOG 169 [70]	GOG 179 [71]	GOG 204 [5]	GOG 240 [57]	JGOG 0505 [6]
Modalities	Cis ± Pac	Cis ± Topo	Cis-Pac	Ctx vs Ctx + bevacizumab	CDDP-Pac vs CB-Pac
			vs Cis- Topo		
			vs Cis- GC		
			vs Cis-VR		
Stage	IVB, recurrent, or persistent SCC	IVB, recurrent, or persistent SCC	IVB, recurrent, or persistent SCC, ACA, or ASC	IVB, recurrent, or persistent SCC, ACA, or ASC	IVB, recurrent, or persistent SCC, ACA, or ASC
N	264	293	513	452	253
PS	0–2	0–2	0–1	0–1	0–2
ORR	19 vs 36 %	13 vs 27 %	29.1 vs 23.4 vs 22.3 vs 25.9 %	36 vs 48 %	-
PFS	2.8 vs 4.8 mo	2.9 vs 4.6 mo	5.8 vs 4.6 vs 4.7 vs 3.9 mo	5.9 vs 8.2 mo	6.9 vs 6.21
*P* value	<001	NS	.06 vs .04 vs .19	.002	.004
OS	8.8 vs 9.7 mo	6.5 vs 9.4 mo	12.8 vs 10.2 vs 10.3 vs 9.9 mo	13.3 vs 17 mo	18.3 vs 17.5 mo
*P* value	NS	.021	.71 vs .90 vs .89	.004	.032

*JGOG* Japanese Gynecologic oncology group; *Cis* cisplatin; *Pac* paclitaxel; *Topo* topotecan; *GC* gemcitabine; *VR* vinorelbine, *CB* carboplatin; *SCC* squamous cell carcinoma; *ACA* adenocarcinoma; *ASC* adenosquamous carcinoma; *N* numbers; *PS* performance status; *ORR* overall response rate; *HR* hazard ratio; *mo* months; *PFS* progression free survival; *NS* non significance; *OS* overall survival

Several questions remain around optimal use of bevacizumab. The JGOG 0505 clinical trial [6] established non-inferiority of the better tolerated combination of carboplatin and paclitaxel compared to cisplatin-paclitaxel. Extrapolating from other gynecologic cancers, bevacizumab should be safe in combination with this regimen in women with cervical cancer, however its efficacy has not been evaluated in a prospective randomized trial. In addition, extrapolating from ovarian cancer, in the real world setting there may be a role for continuing bevacizumab alone following discontinuation of chemotherapy especially given the potential improvement in quality of life from discontinuing cytotoxic agents. More data is required in order to endorse this approach.

Evidence from the GOG 109 [5] and a number of other studies showed that improved oxygenation and tumors with higher MVD can lead to better outcomes with chemoradiotherapy. The combination of an anti-angiogenic agent, that promotes vascular normalization and improved oxygenation combined with multimodality therapy could potentially lead to better outcomes. However, data concerning anti-angiogenic agent/radiotherapy combinations in other tumor types suggest increased risk of fistula formation [72], already a concern in women receiving bevacizumab in recurrent and metastatic disease. Radiation Therapy Oncology Group (RTOG) 1704 evaluated the safety and toxicity profile of adding bevacizumab (10 mg/kg every 2 weeks) for three cycles to pelvic chemoradiotherapy and brachytherapy. In 49 untreated patients with locally advanced cervical cancer (stage IB–IIIB), with a median follow-up of 3.8 years, the 3-year OS was 81.3 % (95 % [CI], 67.2–89.8 %) and the 3-year locoregional failure was 23.2 %. These outcomes compare favorably with historical reports. In addition, the combination was associated with minimal protocol-defined toxicity, the most common toxicity being myelosuppression. Of note, there were no grade 4 gastrointestinal toxicities or gastrointestinal fistulas or perforations [73].

Moving beyond bevacizumab, exploration of novel anti-angiogenic agents targeting parallel angiogenesis related pathways are being undertaken and considered in women with cervical cancer. Single agent, orally administered, multi-TKIs, pazopanib (VEGFR 1, 2, and 3; PDGFR-α and β; and c-KIT inhibitor) and sunitinib (VEGFR 1, 2 and 3; PDGFR, c-KIT, and FLT3 inhibitor) have been investigated. Sunitinib, tested in a phase II clinical trial in patients with unresectable, locally advanced or metastatic cervical carcinoma, was associated with an unacceptably high (26 %) rate of fistula formation combined with only modest activity (no documented objective responses and median time to progression of 3.5 months) therefore further investigation was not warranted [74]. In a second, larger phase II study, 230 patients, with stage IVb persistent/recurrent cervical carcinoma not amenable to curative therapy and at least one prior regimen in the metastatic setting, were randomly assigned to one of three arms: pazopanib alone, lapatinib (a TKI targeting EGFR and HER2/neu) alone, or a combination of the two agents. Pazopanib improved PFS (HR 0.66; 90 % CI, 0.48 to 0.91; *p* = 0.013) and OS (HR 0.67; 90 % CI, 0.46 to 0.99; *p* = 0.045) compared with lapatinib alone. Median OS was 50.7 weeks compared with 39.1 weeks for pazopanib and lapatinib, respectively. Pazopanib alone was well tolerated, but the combination of the two drugs lacked effıcacy and importantly, the combination arm was terminated at the planned interim analysis for futility due to the significant association with more serious adverse events [75]. Recently, the CIRCCa trial presented in the 2014 ESMO Congress [76] evaluated cediranib (AZD2171), a selective, orally bioavailable TKI of VEGFR-1, 2, and 3, in 69 women with primary metastatic or relapsed cervical cancer. In the CIRCCa trial, patients were randomized (1:1) to receive carboplatin, tri-weekly paclitaxel, for a maximum of 6 cycles plus cediranib (20 mg/day) or placebo concurrently with chemotherapy, and later as maintenance therapy until progression. The addition of cediranib improved median PFS by 5 weeks (8.8 vs 7.5 months; *p* = .046) and response rate by 24 % (*p* = .03). However, as CIRCCa closed prematurely owing to the cessation of commercial production of cediranib, the statistical analysis of the difference in median OS between the two groups was underpowered for comparison (59 vs. 63 weeks; HR, 0.93; 80 % CI, 0.64 to 1.36; *p* = 0.401). However, the addition of cediranib significantly increased the rate of diarrhea grades 2–4 (50 % compared with 18 % in the placebo group (*p* = .005) and hypertension (34 v 12 %, *p* = .038, respectively). Brivanib, another TKI which targets VEGFR2 and FGFR-1, is currently being evaluated in a phase II study (NCT01267253) conducted by the GOG.

In addition, non-VEGF-dependent therapeutic approaches, including angiopoietin inhibitors, involve other classes of potentially attractive anti-angiogenic drugs and are under investigation in other tumor types. These should also be explored in cervical cancer patients. Furthermore, given that Ang-2 promotes the proangiogenic action of VEGF, the inhibition of Ang-2 and VEGF together could have complementary actions, thus, the combination of an angiopoietin inhibitor, such as Trebananib (AMG386) and an agent such as bevacizumab could be more active than either agent alone. Combining anti-angiogenic agents with drugs which target the PI3K/AKT/mTOR pathway may also offer an unique treatment opportunity. Finally, the role of immunotherapy in the treatment of cervical cancer is under investigation; potentially combining this approach with an anti-angiogenic agent may represent a novel therapeutic opportunity for this patient population.

As more data emerge about the genomic landscape of cervical cancer and its “potentially druggable” mutations rational combinations with anti-angiogenic agents will potentially be identified. However, as with all rare cancers, it is vital that any studies undertaken have a strong underlying rationale and that they are designed to maximize the biological information we can learn from them. Clearly the way forward to improve outcome for advanced cervical cancer is to reduce the rate of recurrence. We have reached the tolerance of the combination of chemotherapy with radiotherapy in the treatment of locally advanced cervical cancer and the coming generation of trials need to explore the role of targeted therapy in combination with chemoradiotherapy in this setting.

## Conclusions

Despite the introduction of screening and vaccination programs cervical cancer remains a significant health problem. The results from the GOG protocol 240 and the FDA approval of bevacizumab in combination with chemotherapy for the treatment of women with advanced stage, persistent, or recurrent cervical cancer has established the role for new target therapies in a population with historically limited options. However in order to optimize the use of this agent we need to learn more about patients at risk of toxicity and explore opportunities for developing predictive biomarkers. Moving forward there is a very strong rationale for further exploration of angiogenesis pathways alone and in combination in cervical cancer. However, globally we need to advocate for affordable and accessible therapeutic options for women affected by this disease.

## Abbreviations

HPV: Human papillomavirus
VEGF: Vascular endothelial growth factor
US: United States
FIGO: International federation of obstetrics and gynecology
QoL: Quality of life
GOG: Gynecologic oncology group
OS: Overall survival
ORR: Overall response rate
JGOG: Japanese gynecologic oncology group
FDA: Food and drug Administration
HIF-1α: Hypoxia-inducible factor 1 alpha
VEGFR: Vascular endothelial growth factor receptor
PLGF: Placental growth factor
PDGF: Platelet derived growth factor
Ang: Angiopoietins
TKIs: Tyrosine-kinase inhibitors
FGF: Fibroblast growth factor
FGFR: Fibroblast growth factor receptor
PDGFR: Platelet-derived growth factor receptor
HGF: Hepatocyte growth factor
pRb: Retinoblastoma protein
HDAC: Histone deacetylases
TSP-1: Thrombospondin-1
MVD: Microvessel density
PFS: Progression free survival
CD: Cluster of differentiation
IND: Investigational New Drug Application
NSCLC: Non-small cell lung cancer
BC: Breast cancer
RCC: Renal carcinoma
CRC: Colorectal cancer
OC: Ovarian cancer
SD: Stable disease
CI: Confidence interval
HRQoL: Health related quality of life
PD: Disease progression
CR: Complete response
PS: Performance status
m^2^: Square meters
mg: Milligram
iv: Intravenous
Kg: Kilogram
SGO: Society of gynecology oncology (SGO)
HR: Hazard ratio
NCI: National Cancer Institute
DSMB: Data Safety Monitoring Board
PFI: Progression-free interval
FACT-Cx TOI: Functional Assessment of Cancer Therapy—Cervix Trial Outcome Index scale
ICER: Cost-effectiveness ratio
NCCN: National Comprehensive Cancer Network
CTCs: Circulating tumor cells
QALY: Quality adjusted life year
Cis: Cisplatin
Ctx: Chemotherapy
Ifo: Ifosfomide
Pac: Paclitaxel
Topo: Topotecan
GC: Gemcitabine
VR: Vinorelbine
CB: Carboplatin
SCC: Squamous cell carcinoma
ACA: Adenocarcinoma
ASC: Adenosquamous carcinoma
N: Numbers
mo: Months
NS: Non significance
FLT3: Fms-like tyrosine kinase 3
EGFR: Epidermal growth factor receptor
HER2: Human epidermal growth factor receptor 2
